# Immunotherapy – 2075. Towards a pan anti-allergy vaccine

**DOI:** 10.1186/1939-4551-6-S1-P157

**Published:** 2013-04-23

**Authors:** Sari Sabban

**Affiliations:** 1 Molecular Biology and Biotechnology, The University of Sheffield, Sheffield, UK

## Background

Allergy is an increasing autoimmune disease in the developed world. Current consensus is that allergy is the result of a misdirected immune response directed towards ‘seemingly innocuous antigens’. Many of the allergens have similarities with parasite proteins and it is generally accepted that the IgE (Immunoglobulin E) response is a normal defense mechanism against parasitic infestations. The conventional treatment of allergy is through pharmacotherapy where corticosteroids are used to reduce the expression of inflammatory proteins; although effective, they do not combat the underlying cause of allergy. Novel immunotherapeutic treatments have been developed that immunize sufferers with anti-IgE antibodies, but this passive immunization strategy is only temporary, and patients need to return for follow-up injections.

## Methods

This project reports the development of an active anti-allergy immunotherapy. The synthetic peptide 2Fcε_2-3_, derived from human IgE, was injected into rats, and followed up by a boost with a chimeric human-dog-human IgE, in an attempt to direct the immune system and develop polyclonal antibodies that target a certain epitope in the IgE antibody which would lead to the removal of serum and FcεRI receptor bound IgE.

## Results

The experiment resulted in a rat serum with strong antibody titer targeting the 2Fcε_2-3_ peptide, this serum was also found to target native human IgE, as well as the dog and the horse native IgE antibody. Further analysis showed that the serum antibodies cross linked receptor bound IgE and resulted in cell degranulation and mediator release.

## Conclusions

The immunization strategy was successful but did not fully work as predicted. The study does, however, lay significant foundations for future potential anti-allergy vaccine designs.

